# Comparative performance of tuberculin and defined-antigen cocktails for detecting bovine tuberculosis in BCG-vaccinated cattle in natural settings

**DOI:** 10.1038/s41598-025-85389-1

**Published:** 2025-02-07

**Authors:** Abebe Fromsa, Andrew J. K. Conlan, Sreenidhi Srinivasan, Balako Gumi, Wegene Bedada, Miserach Zeleke, Dawit Worku, Matios Lakew, Biniam Tadesse, Berecha Bayissa, Asegedech Sirak, Musse Girma Abdela, Getnet Abie Mekonnen, Tesfaye Chibssa, Maroudam Veerasami, Gareth J. Jones, H. Martin Vordermeier, Nick Juleff, James L. N. Wood, Gobena Ameni, Vivek Kapur

**Affiliations:** 1 Aklilu Lemma Institutes of Pathobiology, Addis Ababa University, Addis Ababa, Ethiopia; 2 College of Veterinary Medicine and Agriculture, Addis Ababa University, Bishoftu, Ethiopia; 3 Disease Dynamics Unit, Department of Veterinary Medicine, University of Cambridge, Cambridge, UK; 4 Huck Institutes of Life Sciences, The Pennsylvania State University, University Park, PA USA; 5 Department of Animal Science, The Pennsylvania State University, University Park, PA USA; 6 The Global Health Initiative, Henry Ford Health, Detroit, MI USA; 7 Animal Health Institute, Sebeta, Ethiopia; 8 CisGen Biotech Discoveries Pvt Ltd, Chennai, India; 9 Animal and Plant Health Agency, Weybridge, UK; 10 Woking, Surrey UK; 11 Department of Veterinary Medicine, College of Agriculture and Veterinary Medicine, United Arab Emirates University, Al Ain, UAE; 12 The Bill & Melinda Gates Foundation, Seattle, WA USA; 13 Present Address: National Veterinary Institute, Bishoftu, Ethiopia

**Keywords:** BCG vaccination, Bovine tuberculosis, Defined antigen cocktails, Tuberculin, IGRA and skin tests, Immunology, Microbiology, Diseases

## Abstract

Bovine tuberculosis (bTB) is a threat to cattle health and public safety. The current control programs are hampered by wildlife reservoirs and socioeconomic barriers. Vaccinating cattle with Bacillus Calmette-Guérin (BCG) effectively reduces transmission, offering a potential solution for controlling bTB. A key requirement for vaccination strategies using BCG is the validation of defined antigens to differentiate infections among vaccinated animals (DIVA). We compared tuberculin with DIVA peptide cocktails (ESAT-6, CFP-10, and Rv3615c) in 67 unvaccinated and 67 BCG-vaccinated cattle exposed to *M. bovis* in a natural setting. The cattle were tested every 4 months with a skin test and every 2 months with interferon-gamma (IFN-γ) release assays (IGRA) over a year of exposure. Before exposure, the DIVA skin, DIVA IGRA, and tuberculin tests showed 100% specificity in unvaccinated control calves. After exposure, the DIVA skin, DIVA IGRA, and comparative cervical tuberculin (CCT) tests had comparable sensitivities of 46% (95% CI 36, 56), 45% (95% CI 35, 55), and 47 (95% CI 37, 57), respectively, when assessed against animals positive by *M. bovis* culture PCR. The results suggest that test-and-slaughter control strategies using tests with low sensitivity are not expected to be effective in controlling bTB in high-prevalence herds, and highlight an urgent need to improve the sensitivity of diagnostic tests for bTB in these settings.

**Supplementary Information:**

The online version contains supplementary material available at 10.1038/s41598-025-85389-1.

## Introduction

Bovine tuberculosis (bTB) is a chronic disease of cattle primarily caused by members of the *Mycobacterium tuberculosis* complex (MTBC), which can infect a wide range of domestic and wild animal species, including humans, posing a significant public health risk in regions with high prevalence^1^. The prevalence of bTB varies by country, influenced by control measures, animal movements and wildlife reservoirs^2^. In Ethiopia, bTB is more prevalent in genetically improved cattle raised for high milk production in intensive farming systems, with prevalence rates of 21.6% in Holstein-Friesians and 9.9% in crossbred cattle^3^. A recent study reported 54.4% of 299 dairy herds and 24.5% of 5,675 cattle were reactive to the comparative intradermal cervical tuberculin test^4^. In the absence of feasible bTB control options, efforts to increase milk productivity through cross-breeding and intensification of cattle farming are likely to increase disease incidence.

Currently, control of bTB relies on tuberculin testing of herds and culling positive test reactors. This approach has successfully reduced the prevalence of the disease in many countries and has led to elimination in Australia^5^. However, this strategy is extremely costly and unaffordable for resource-constrained countries like Ethiopia. It is also less effective in countries where wildlife reservoir hosts are present^6,7^. Bacillus Calmette-Guérin (BCG) vaccination offers a potentially practical and affordable alternative for countries lacking existing control programs^8–10^. Experimental investigations and field studies have demonstrated that while BCG does not protect all vaccinated animals from infection, it significantly reduces the severity of bTB pathology^7,8,10,11^. The BCG vaccine developed from attenuated *M. bovis* and approved for human tuberculosis, is considered a viable candidate vaccine for cattle. However, the World Organization for Animal Health (WOAH) does not recommend BCG for livestock due to its interference with tuberculin-based tests leading to false-positive reactions in official bTB control programs^1^. To overcome the issue of non-specific tuberculin reactions caused by BCG vaccination, researchers are exploring the use of defined antigens that can differentiate infected among vaccinated animals (DIVA), a crucial tool for effective implementation of BCG vaccination as a method of disease control^12,13^.

Comparative genomic and transcriptomic studies have identified several antigens present in *M. bovis* field strains but either absent or not immunogenic in the BCG vaccine strain, enhancing the specificity of tuberculin-based bTB diagnostic tests^14,15^. Previous studies have shown that recombinant peptide antigens induce a greater skin response when used in combination than when used alone^16^. Notable candidate antigens that demonstrated significant DIVA capability include ESAT-6, CFP-10, and Rv3615c, indicating a potential path to use along with BCG vaccination for bTB management and control^13,17^.

The performance of these antigens is subject to variability depending on the geographical conditions, prevailing *Mycobacterium* lineages, dosage, cutoff values, and features of the examined population. Variability in the levels of interferon-gamma (IFN-γ) and innate immune response among different strains of *M. bovis* has been reported^18^. Therefore, prior to practical deployment, thorough validation of these DIVA antigens is required across a varied range of cattle breeds and geographic regions to determine their specificity and sensitivity. However, existing research data on the performance of DIVA tests in BCG-vaccinated animals are limited to studies primarily performed in the UK under experimental transmission^19,20^, which might not reflect the performance of DIVA antigens in naturally infected cattle. Although other studies have also tested DIVA and tuberculin in BCG-vaccinated and unvaccinated cattle under natural transmission settings, they were limited to evaluating the efficacy of the BCG vaccine^7,8,21–23^ or comparing the relative performance of antemortem tests without postmortem confirmation^13,17,24^.

This study examined diagnostic data collected for the evaluation of BCG efficacy via a natural transmission experiment^10^ for the assessment of the immunological responses of BCG-vaccinated cattle to mycobacterial antigens. The other similar studies published thus far have relied on the use of the DIVA skin test and IFN-γ release assay (IGRA) to determine the infection status of artificially challenged animals based on predetermined cutoff values. Here, we compared the quantitative responses of vaccinated and control groups to DIVA antigens against tuberculin. The study animals were followed longitudinally, with repeated tests and postmortem confirmation of infection status, providing a unique opportunity to assess the performance of bTB diagnostic tests in animals with confirmed (postmortem) disease status. Here, we evaluated the immune responses of DIVA peptide antigen cocktails on 67 BCG-vaccinated and 67 unvaccinated calves via both skin tests and in vitro IGRA formats and compared the performance of these peptides to that of tuberculin.

## Materials and methods

### Study setting

The study took place at the Animal Health Institute (AHI) in Sebeta, which is located approximately 20 km southwest of the capital city, Addis Ababa, Ethiopia. At the AHI premises, three separate barns were constructed. Each barn had the capacity to accommodate 80 adult cattle and had a fenced outdoor area measuring approximately 2,000 to 2,500 m^2^. These barns were approximately 300 m apart from each other. Two of the fenced barns were designated for housing the experimental replicate groups (seeders and sentinels) throughout the entire exposure period. This was done to control for any potential variations in exposure resulting from differences in infectiousness among the seeder animals. The third barn was used to house and acclimate the sentinel calves immediately after they were recruited and before they were exposed to the seeders. To ensure proper waste management, septic tanks were constructed behind each barn. The feces and urine were directed into the septic tanks through large pipes that connected the barns to the exterior premises. The animal attendants were all educated about the zoonotic risk of bTB. They were required to wear personal protective equipment, including N95 masks, disposable gloves, and rubber boots, whenever they handled animals or worked inside the barns. All relevant local biosecurity and safety procedures were strictly adhered to.

### Study animals and design of the experiment

**Fig. 1:**
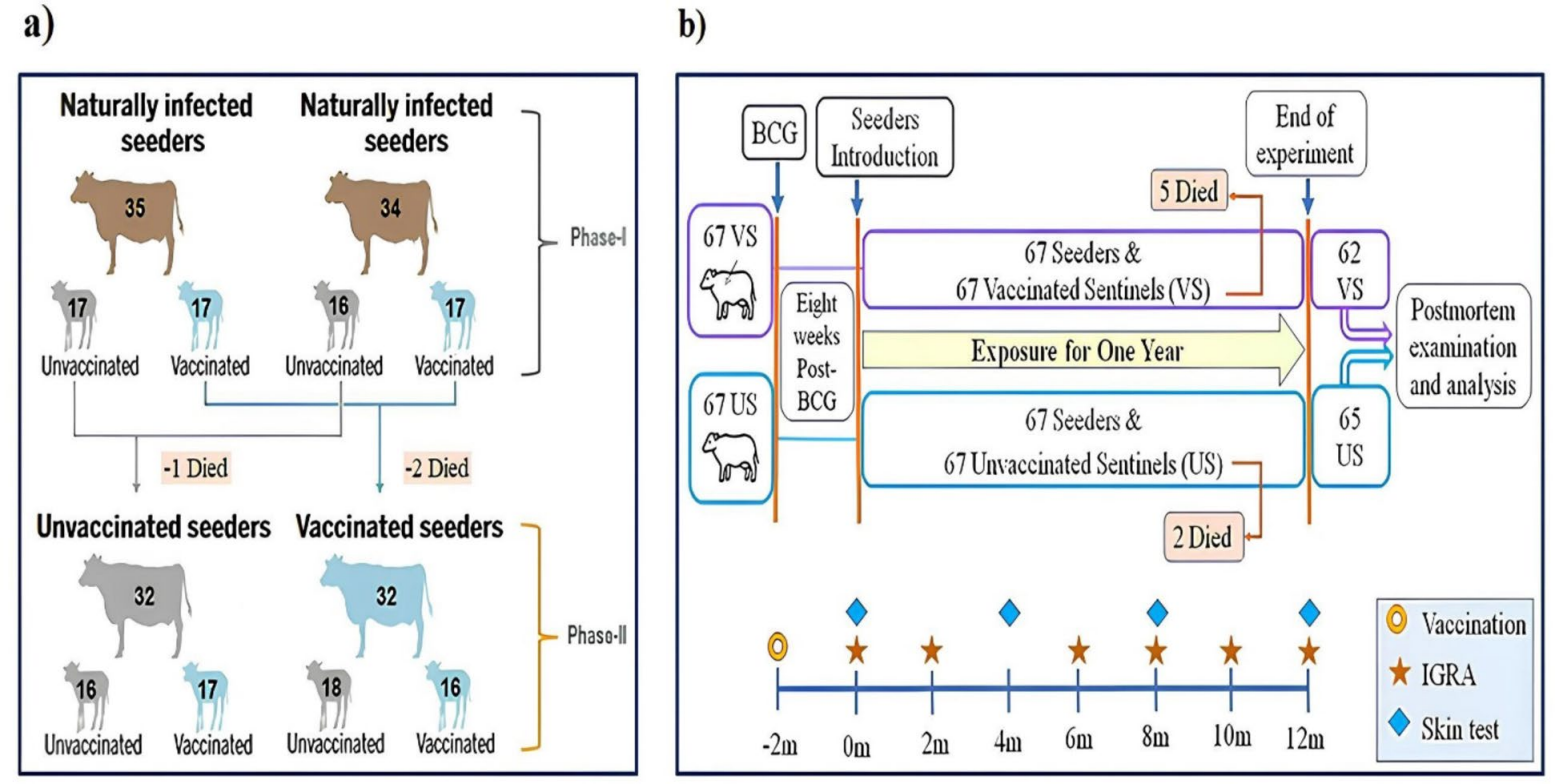
Schematic representation of the experimental protocol. (**a**) The two-phase cross over design of the experiment and (**b**) Testing schedules and postmortem examination. In phase I, two groups (Groups 1 & 2) functioned as biological replicates and were kept in a separate barn (**a**) Phase I, Group 1 (top left): 17 unvaccinated controls and 17 vaccinated calves were exposed to 35 seeders. Phase I, Group 2 (top right): 16 unvaccinated controls and 17 vaccinated calves were exposed to 34 seeders. Phase II, Group 1 (lower left): the surviving 32 unvaccinated control sentinels from Phase I were pooled and became seeders for 16 unvaccinated controls and 17 vaccinated calves. Phase II, Group 2 (lower right): the surviving 32 vaccinated calves from Phase I were pooled and became seeders for 18 unvaccinated controls and 16 vaccinated calves. (**b**) From both Phase-I and Phase-II, a total of 67 unvaccinated control (67 U) and 67 vaccinated (67 V) calves were exposed to their respective seeders eight weeks after vaccination and kept in contact for twelve months. During the twelve-month contact period, 5 vaccinated calves and 2 unvaccinated controls died across both phases, resulting in 62 vaccinated and 65 unvaccinated controls reaching the endpoint. IGRA tests were performed every two months, and skin tests were conducted every four months, starting just before exposure (0 m) and continuing until the end of the experiment (12 m). At the end of the experiment, all animals were slaughtered, and a postmortem examination was conducted to detect bTB lesions, followed by mycobacterial culture and PCR testing of culture-positive isolates.

A total of 72 adult cattle, naturally infected and identified as reactors through skin and blood tests, were selected as seeders for the natural transmission experiment. The primary objective of our experimental study was to assess the efficacy of BCG vaccination through a natural transmission experiment. In this report, we conducted a secondary analysis of diagnostic data collected from the BCG efficacy study^10^. The experiment was divided into two phases, each involving an equal number of calves and comprising two biological replicate groups housed in separate barns. The reason for conducting the study in two phases was to measure both how well the vaccine prevented infection (direct effect) and how it reduces the infectiousness of infected vaccinated animals (indirect effect) for a more complete understanding of the total efficacy of the BCG vaccine. To find naive sentinels, dairy herds were screened using both skin tests and IGRA. For each phase, 68 male calves, less than three months old, were recruited from dairy herds that tested negative for bTB. These herds were located in Koka, Ziway, Alage, Hawassa, Wondogenet, Jimma, and Ambo towns. The recruited sentinels were housed separately from the seeders to prevent early exposure. After an acclimatization period of three to twelve weeks, the sentinels that tested negative in the bovine (PPD-B) minus avian (PPD-A) tuberculin IGRA test were randomly assigned to either the control or vaccinated groups using a double-blind lottery system. Calves with a positive OD450 nm ≥ 0.1 on the bovine minus avian tuberculin IGRA test were excluded. In the first phase, 33 unvaccinated controls and 34 vaccinated calves were included. In the second phase, 34 unvaccinated controls and 33 vaccinated calves were included, resulting in a total of 67 calves per group across both phases. During the trial period, calves were fed boiled and pasteurized milk until they reached six months of age. Afterward, weaned calves and adult seeders were provided with concentrate feed, hay, green fodder, and water *ad libitum*. Throughout the twelve-month contact period, 5 vaccinated calves and 2 unvaccinated controls from both phases died, resulting in 127 (62 vaccinated and 65 unvaccinated controls) reaching the endpoint (Fig. 1).

### Test antigens

The PPD (purified protein derivative) tuberculin were procured from Prionics Lelystad BV, Lelystad, the Netherlands. DIVA peptide cocktails consisting of peptides representing ESAT-6, CFP-10, and Rv3615c were commercially synthesized and previously described^13^.

### BCG vaccination

The vaccine was supplied as freeze-dried preparation and reconstituted in Sauton’s medium as per the manufacturer’s instruction (Green Signal Bio Pharma, Pvt. Ltd., India) in the first phase and (AJ Vaccines A/S, Artillerivej 5, DK 2300 Copenhagen S, Denmark) in the second phase. The vaccinated group of calves were administered a subcutaneous injection of 0.5 ml (1–4 × 10^6^ CFU) of live attenuated BCG Danish Strain SSI 1331 (Staten’s Serum Institute [SSI], Copenhagen, Denmark). Conversely, the control group received 0.9% normal saline. A total of 67 calves received the BCG vaccination, while the remaining 67 sentinel calves were not vaccinated. The authors conducting the field work were blinded to the vaccination status of the animals until the conclusion of the experiment.

### Skin and IGRA testing schedules

The sentinel animals underwent skin testing every four months and IGRA every two months using DIVA, avian, and bovine tuberculin antigens in both the skin and IGRA test formats (Fig. 1). The skin test and IGRA results analyzed and reported here are from the 12th -month post-exposure test, except for Table 1; Fig. 1, which are based on pre-exposure test result and the Supplementary Fig. S1, which includes tests of all postexposure-time points.

**Table 1:** The specificity of the DIVA and tuberculin elicited IFN-γ and skin test responses eight-week post BCG vaccination.

Test	Antigen	Group	Test status		Specificity% (95% CI)	P value
			Positive	Negative		
IGRA	DIVA	Control	0	67	100 (93.5, 100)	> 0.99
		Vaccinate	1	66	98.5 (91.3, 100)	
	PPD(B-A)	Control	7	60	89.6 (79.7, 95.1)	**< 0.01**
		Vaccinate	30	37	**55.2** (43.4, 66.5)	
	PPD-B	Control	24	43	64.2 (52.2, 74.6)	**< 0.01**
		Vaccinate	48	19	28.4 (18.9, 40.2)	
Skin	DIVA	Control	0	67	100 (93.5, 100)	0.12
		Vaccinate	4	63	94.0 (85.2, 98.1)	
	CCT	Control	0	67	100 (93.5, 100)	**< 0.01**
		Vaccinate	22	45	**67.2** (55.2, 77.3)	
	SIT	Control	0	67	100 (93.5, 100)	**< 0.01**
		Vaccinate	53	14	**20.9** (12.8, 32.2)	

The cutoff value for all antigens tested in the IFN-γ assay was ≥ 0.1, whereas for the skin test, the cutoff values used were > 4 mm for the CCT, ≥4 mm for the SIT and ≥ 2 mm for the DIVA.

*CCT* comparative cervical tuberculin test, *PPD* purified protein derivative tuberculin, *PPD-B* bovine tuberculin, *PPD (B-A)* bovine minus avian tuberculin, *SIT* single intradermal tuberculin test.

Significant values are in bold.

### Skin testing procedure

For the skin test, hair was shaved at two sites on the right and one site on the left mid-cervical area. Bovine tuberculin (30,000 IU/ml), avian tuberculin (25,000 IU/ml), and the DIVA peptide cocktail (100 µg/ml of each peptide) were injected intradermally in 0.1 mL volumes. Skin fold thickness was measured to the nearest millimeter using Irish calipers by the same person before and 72 h after antigen administration. The change in skin thickness was determined by subtracting the pre-injection measurement from the 72-hour post-injection measurement for each antigen. The cut-offs used to determine test status were > 4 mm for the Comparative Cervical Tuberculin (CCT), ≥ 4 mm for the Single Intradermal Tuberculin Test (SIT), and ≥ 2 mm for the DIVA antigens.

### IGRA testing procedure

The IGRA test was conducted using the BOVIGAM^®^ kit (Thermo Fisher Scientific Inc., USA). Approximately 5 ml of blood was collected from each animal’s jugular vein in lithium heparin vacutainers. Duplicate wells were stimulated with 25 µl of each antigen and 250 µl of whole blood, resulting in final concentrations of 1 µg/ml DIVA antigens, 250 IU/ml avian tuberculin (PPD-A), and 300 IU/ml bovine tuberculin (PPD-B). Positive and negative controls were stimulated with pokeweed mitogen (PWM) at 10 µg/ml and RPMI (Nil), respectively. Samples were incubated at 37 °C in a humid atmosphere for 18–24 h. The culture supernatants from duplicate samples were pooled and stored at – 80 °C until the IFN-γ test was performed. The results of the IGRA test were reported as the change in optical density (OD) at 450 nanometers (ΔOD450 nm). This was determined by subtracting the OD450 nm of whole blood cultures stimulated with antigen from the OD450 nm of cultures without antigen. Optical Density (OD) refers to the amount of light absorbed by the sample, and 450 nm is the specific wavelength of light used for the measurement. The intensity of the color corresponds to the OD450 nm value and is directly related to the concentration of the target antigen in the sample.

### Postmortem examination

After humanely euthanizing the animals using a captive bolt (CASH^®^ special 0.22 caliber penetrating captive bolt) followed by immediate exsanguination, postmortem examinations were carried out by a skilled pathologist assisted by two veterinarians with extensive experience in diagnosing bTB lesions. At the end of the natural exposure to infection, all calves were euthanized and examined. Following visual inspection and palpation of each lymph node and tissue in the carcass, the relevant organs for sampling were separated and transferred to the postmortem examination table for detailed evaluation. To assess the true disease status, all the lymph nodes were sliced into 1–2 cm thick sections and meticulously examined. The animal was classified as bTB lesion positive (lesioned) if any of the examined tissues had gross visible lesions consistent with bTB and negative (non-lesioned) if no obvious lesions were found. The severity of the gross lesions was evaluated using the semiquantitative technique developed by Vordermeier et al.^25^ taking the number and size of the observed lesions into account.

### Tissue sampling for mycobacterial culture

For bacterial culture, samples were collected from ten different tissues of each animal, including mandibular, lateral retropharyngeal, medial retropharyngeal, bronchial, cranial tracheobronchial, mediastinal, hepatic and mesenteric lymph nodes as well as from the left and right lung lobes, regardless of the presence or absence of lesions. Extra samples were collected when lesions were found in other examined lymph nodes and tissues. The lymph node and tissue samples were collected in sterile 50 mL Falcon tubes containing 10–15 mL of 0.9% saline solution and transported to the laboratory for bacterial culture and isolation. Prior to processing, the tissue samples were frozen at − 20 °C.

### Mycobacterial culture

For mycobacterial isolation, lymph nodes or tissue samples were cut with sterile blades into thin slices. The pieces were then homogenized with a sterile pestle and mortar and transferred into a sterile plastic bag for further grinding in a stomacher. Then, 5 ml of the homogenate was transferred to a sterile 50 ml Falcon tube, decontaminated by adding an equal volume of 4% NaOH and mixing for 15 min. The mixture was neutralized by adding 40 ml of PBS, followed by centrifugation at 1,106 g for 15 min. The supernatant was discarded, and 0.1 ml of the sediment from each sample was spread onto duplicate slants of Lowenstein–Jensen (LJ) medium (Sigma Aldrich, Co, St. Louis, USA)^1^, one enriched with sodium pyruvate and the other with glycerol. The cultures were then incubated aerobically at 37 °C for up to 8 weeks, after which the growth of the mycobacterial colonies was observed weekly. Slants that did not show growth beyond the 8th week were considered to be negative. Ziehl–Neelsen (ZN) staining technique was used to confirm the presence of acid-fast bacilli (AFB) in the smears of culture-positive colonies.

### DNA extraction

DNA was extracted from culture isolates using the DNeasy Blood and Tissue Kit (QIAGEN) with some modifications. First, loopfuls of cells were removed from culture-positive LJ media using a sterile loop and transferred to 2 ml Eppendorf tubes containing 300 μl of TE buffer. The tubes were then incubated at 85 °C for 60 min on a heating block to inactivate the bacteria. Lysis buffer was prepared by adding 20 ml of 20 mm Tris-HCl, 12 ml of Triton X and 2 ml of EDTA to one liter of water, after which the mixture was stored at room temperature. Immediately before use, 80 mg of lysozyme was added per ml of lysis buffer to make a working lysis buffer solution. For each reconstituted culture sample, 100 µl of working lysis buffer solution was added. The samples were incubated at 37 °C for 1 h. Then, 180 µl of ATL buffer and 20 µl of proteinase K were added to each sample. The samples were incubated at 56 °C for 3 h and vortexed every hour to ensure complete mixing. Then, all downstream procedures were performed using the reagents provided by the manufacturer as described in QIAGEN^26^, and the DNA was eluted in 100 µl of AE buffer.

### PCR (polymerase chain reaction)

The DNA samples were amplified by two-step real-time multiplex PCR at the AHI, Sebeta, Ethiopia. In the first step of the multiplex PCR cycle, three primer-probe sets targeting IS1081, the yrbE3A gene and the ‘RD7’ deletion were used for the collective identification of both animal- and human-adapted, animal-adapted and human-adapted MTBC species. In the second step, three additional primer sets targeting the RD4 gene deletion, SNP at the lepA locus and the SNP at the rskA locus were used for specific identification of *M. bovis*,* M. caprae and M. origis*, respectively. The target genes and primer/probe set sequences used in the current multiplex PCR assay are as describe in our pervious study^27^.

The amplification was carried out in a 20 µL final reaction mixture, which consisted of 5 µL of DNA template, 10 µL of prime gene expression master mix with ROX dye, 0.5 µL of each primer, and 3.5 µL of nuclease-free water. We used nuclease-free water as the negative control and synthetic gDNA targets (IDT, Iowa, USA) as the external positive controls in duplicate for each PCR run to ensure that the master mix and amplification conditions worked as expected. The IS1081 sequence, a common target for all MTBCs, was used as an internal amplification control. The results were considered invalid if the positive control was not detected or if the negative control tested positive. Amplification was performed using the QuantStudio™ 6 Flex Real-Time PCR System (Applied Biosystems Life Technologies, Thermo Fisher Scientific, Waltham, MA, USA). The thermocycler protocol included one cycle of 3 min at 95 °C for polymerase activation, 40 cycles of 15 s at 95 °C for denaturation, and 40 cycles of 60 s each at 63 °C for annealing/extension steps. For all primer-probe sets, a sample was considered PCR positive when the cycle threshold (Ct) value was ≤ 40.

### Ethics statement

Prior to commencing the study, ethical approval was granted by the Institutional Review Board of Aklilu Lemma Institute of Pathobiology (reference number ALIPB IRB/44/2013/21). The experiment was conducted according to the Technical Guideline for National Research Ethics Review, ensuring compliance with the guidelines^28^. Additionally, the study adhered to and reported following the ARRIVE guidelines 2.0.

### Statistical analyses

Data analysis was performed using GraphPad Prism version 9.4.0 for Windows (GraphPad Software, San Diego, California, USA), the R statistical language^29^ and R Studio^30^. Plots were produced using the ggplot2^31^ and ‘ggstatsplot’ packages^32^. Normality tests were conducted using the Shapiro-Wilk test with the normality() function from the {dlookr} package in R. Additionally, visual inspection of the distributions was conducted using the plot_histogram() and plot_density() functions from the {DataExplorer} package in R. Based on the results, the nonparametric statistical tests were used: the Wilcoxon rank-sum (Mann-Whitney U) test was applied to determine differences in skin and IFN-γ test responses between the two treatment groups; the nonparametric Friedman repeated measures ANOVA with Bonferroni correction for multiple comparisons was used to determine differences in skin and IFN-γ responses between different antigens within a group and the nonparametric Spearman’s rank correlation coefficient was used to determine the strength and direction of association between the different test antigens IFN-γ and skin test responses. The rank biserial correlation coefficients were calculated using the ggbetweenstats() and ggwithinstats() functions from the {ggstatsplot} package in R. The ggbetweenstats() function was used for comparisons between two independent groups (vaccinate vs. control response to each antigen) while the ggwithinstats() function was used for comparisons involving more than two dependent groups (animal immune response to different antigens). Sensitivity, specificity, positive likelihood ratio (LR+), negative likelihood ratio (LR−), and accuracy were calculated using 2 × 2 contingency tables (see Supplementary Table S1). In the contingency tables, columns indicate the disease status (positive or negative) determined by *M. bovis* PCR (Table 3) and visible lesions, culture or *M. bovis* PCR (Supplementary Table S2) used as a reference standard. The rows represent the positive or negative results obtained using the antemortem tests. The cells labeled (a) and (d) represent true positive and true negative test results, respectively while the cells labeled (b) and (c) represent false positive and false negative test results, respectively. The measures of diagnostic test performance were calculated as follows: sensitivity = a/(a + c); specificity = d/(b + d); LR+ = (a/(a + c))/(b/(b + d)); LR− = (c/(a + c))/(d/(b + d)); and overall diagnostic accuracy = (a + d)/N^33–35^. Cohen’s kappa statistic, used to measure binary agreement between two tests for classifying animals into positive or negative groups, was calculated using the free online Graphpad web calculator. For statistical significance, *P* < 0.05 and 95% CI were used.

### Definitions and interpretation of diagnostic test performance measures

Sensitivity refers to the proportion of infected animals (true positives) that are correctly identified by the test, while specificity refers to the proportion of animals without the disease that the test correctly identifies as negative. A high sensitivity indicates that the test effectively identifies infected animals, reducing the risk of false negatives, while a high specificity indicates that the test effectively excludes non-infected animals, reducing the risk of false positives. The overall accuracy of a test refers to the proportion of correct identifications computed by dividing the sum of true positives and true negatives by the total number of animals tested^35^. The LR+ indicates the likelihood of observing a positive test in animals with the disease compared to those without the disease. A favorable test has a high positive likelihood ratio. The negative likelihood ratio (LR−) reflects how much less likely a negative test result is in diseased animals compared to healthy ones. A low LR− means that the disease is unlikely, with a negative test. The lower the value of (LR−), the more valuable that test is for increasing certainty about a negative diagnosis^35,36^. Both LR+ and LR− close to 1.0 suggest that the test does not distinguish between animals with or without the disease^35^. Kappa was interpreted using a scale as follows: kappa ≤ 0 as indicating no agreement, 0.01–0.20 as none to slight, 0.21–0.40 as fair, 0.41–0.60 as moderate, 0.61–0.80 as substantial, and 0.81-1.00 as almost perfect agreement^37^.

## Results

### Skin test and IGRA responses of experimental calves’ post BCG vaccination prior to exposure to infected animals

**Fig. 2:**
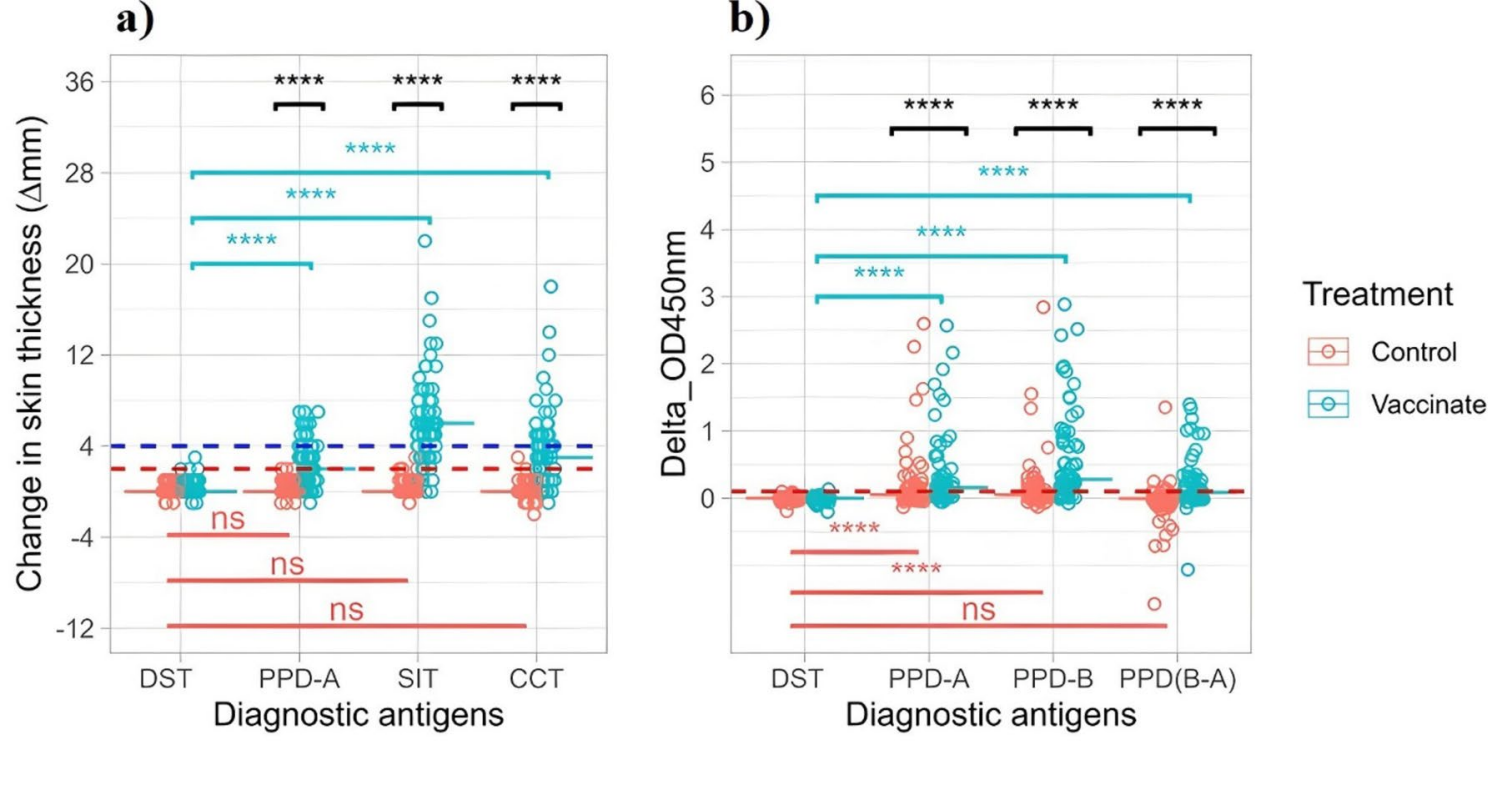
Eight-week post-BCG immune response to tuberculin and DIVA antigens. (**a**) changes in skin thickness and (**b**) IFN-γ responses to DIVA and tuberculin in 67 BCG-vaccinated and 67 unvaccinated naïve calves. Skin and IFN-γ test responses to DIVA, avian, and bovine tuberculin were evaluated 8 weeks after BCG vaccination. The dotted blue horizontal lines at 4 mm and the dotted red horizontal lines at 2 mm are the cutoffs used for the tuberculin and DIVA skin test antigens, respectively. The dotted red horizontal line at 0.1 is the cutoff values used for all tuberculin and DIVA IFN-γ antigens, and the horizontal lines within the plots of each treatment group represent the median response to all the respective antigens. The statistical significance of differences in skin reaction and IFN-γ production between different antigens within a treatment group were determined using Friedman nonparametric analysis of variance with Bonferroni correction for multiple comparisons. Statistical significance of differences between the control and vaccinated groups were determined using the Wilcoxon rank-sum (Mann‒Whitney U) test. (****P < 0.0001; *ns* not significant (*P* > 0.05)).

DIVA skin and all tuberculin skin test antigens elicited no positive responses in the unvaccinated control group, demonstrating 100% specificity within this sample (Fig. 2a; Table 1). The DIVA skin test was positive for four vaccinated animals (specificity 94.0 (95% CI: 85.2, 98.1) (Table 1). Three DIVA skin test-positive vaccinated calves showed a 2 mm increase in skin thickness, while one showed a 3 mm increase at pre-exposure. However, at the fourth month post-exposure, only one showed a 2 mm increase, while the other three showed no change, with two consistently testing negative throughout the exposure period. Statistically no significant difference was observed between the proportions of vaccinated and control animals that responded positively to DIVA skin test (*p* = 0.12) (Table 1). However, only one vaccinated calf exhibited a positive response to the DIVA IFN-γ assay (specificity 98.5% [95% CI 91.3, 100]). Although the OD450 nm value of the positive IFN-γ response in this vaccinated calf was 0.1, which falls on the positive cutoff value of the test (Fig. 2b), the animal tested negative (∆OD450 nm = 0.00) on the same assay at the two months after exposure. In contrast, the proportion of vaccinated animals that responded positively to the tuberculin skin test and IFN-γ assay was significantly greater than that of the controls (*p* < 0.001), confirming the lack of specificity of tuberculin antigens (Table 1).

Normality tests conducted on all measured antigen test responses resulted in p-values of < 0.0001, and visual inspection of the distributions confirmed their non-normality with evident asymmetry, dictating non-parametric statistical tests for comparing responses between treatments and antigens. Eight weeks after vaccination, the BCG-vaccinated calves showed a significant increase in skin fold thickness and IFN-γ response to all tuberculin antigens compared to those of the unvaccinated controls (Fig. 2a and b). However, for DIVA IGRA test, there was no statistically significant difference in IFN-γ production between the two groups, as indicated by a p value of 0.79. The rank biserial correlation coefficient (r) was close to zero (0.03), suggesting a negligible effect of BCG vaccination on IFN-γ production response to DIVA-specific antigen (Fig. 2a and b).

Friedman repeated measure nonparametric ANOVA with Bonferroni correction for multiple comparisons used to compare the responses to different antigens showed that all tuberculin elicited significantly greater skin and IGRA (*p* < 0.001) test responses than DIVA antigens in BCG-vaccinated animals. In contrast, in the controls, the DIVA and all tuberculin antigens elicited similar and low skin test responses (Fig. 2a). In the unvaccinated controls, avian and bovine tuberculin induced a significantly higher IFN-γ response than the DIVA. However, there was no significant difference between the DIVA and bovine minus avian tuberculin IFN-γ responses (*P* > 0.05) (Fig. 2b).

### Skin and IGRA responses in experimental calves postexposure to infected cattle

Out of 112 animals with visible lesions, 54 (48%) tested positive for DIVA skin, 49 (44%) for DIVA IGRA, and 49 (44%) for CCT. Similar DIVA skin, CCT and DIVA IGRA tests positive rates were observed in *M. bovis* culture PCR-positive animals (Table 2). These findings suggest consistent and similar accuracy for DIVA skin, DIVA IGRA, and CCT tests in classifying cattle with confirmed *M. bovis* infection by culture PCR and cattle with lesions suggestive of disease (Table 2). On the other hand, bovine minus avian tuberculin IGRA and bovine tuberculin (SIT) skin tests demonstrated higher sensitivity closer to 60% or higher in both confirmed *M. bovis* infection cases and cattle with lesions (Table 2).

**Table 2:** Positive skin and IGRA test responses of experimental calves at the 12th month postexposure to infected cattle in *M. Bovis* PCR, and gross VL test status at PM.

PM tests	Test status	Treatment	N	Tests and number (%) positive					
				Skin test			IGRA test		
				DIVA	CCT	SIT	DIVA	PPD (B-A)	PPD-B
*M. bovis* PCR	Negative	Control	18	7 (39)	5 (28)	10 (56)	3 (17)	9 (50)	17 (94)
		Vaccinate	20	8 (40)	6 (30)	9 (45)	5 (25)	11 (55)	17 (85)
		Subtotal	**38**	**15 (39)**	**11 (29)**	**19 (50)**	**8 (21)**	**20 (53)**	**34 (89)**
	Positive	Control,	47	25 (53)	23 (49)	32 (68)	26 (55)	39 (83)	42 (89)
		Vaccinate	42	16 (38)	17 (40)	30 (71)	16 (38)	29 (69)	38 (90)
		Subtotal	**89**	**41 (46)**	**40 (45)**	**52 (58)**	**42 (47)**	**68 (76)**	**80 (90)**
Gross VL	Negative	Control	4	0 (0)	0 (0)	0 (0)	0 (0)	0 (0)	4 (100)
		Vaccinate	11	2 (18)	2 (18)	5 (45)	1 (9.1)	4 (36)	10 (91)
		Subtotal	**15**	**2 (13)**	**2 (13)**	**5 (33)**	**1 (13)**	**4 (7)**	**14(93)**
	Positive	Control	61	32 (52)	28 (46)	42 (69)	29 (48)	48 (79)	55 (90)
		Vaccinate	51	22 (43)	21 (41)	34 (67)	20 (39)	36 (71)	45 (88)
		Subtotal	**112**	**54 (48)**	**49 (44)**	**76 (68)**	**49 (44)**	**84 (75)**	**100 (89)**

*PCR* polymerase chain reaction, *PM* postmortem, *VL* visible lesion.

Subtotal values are in bold.

**Fig. 3:**
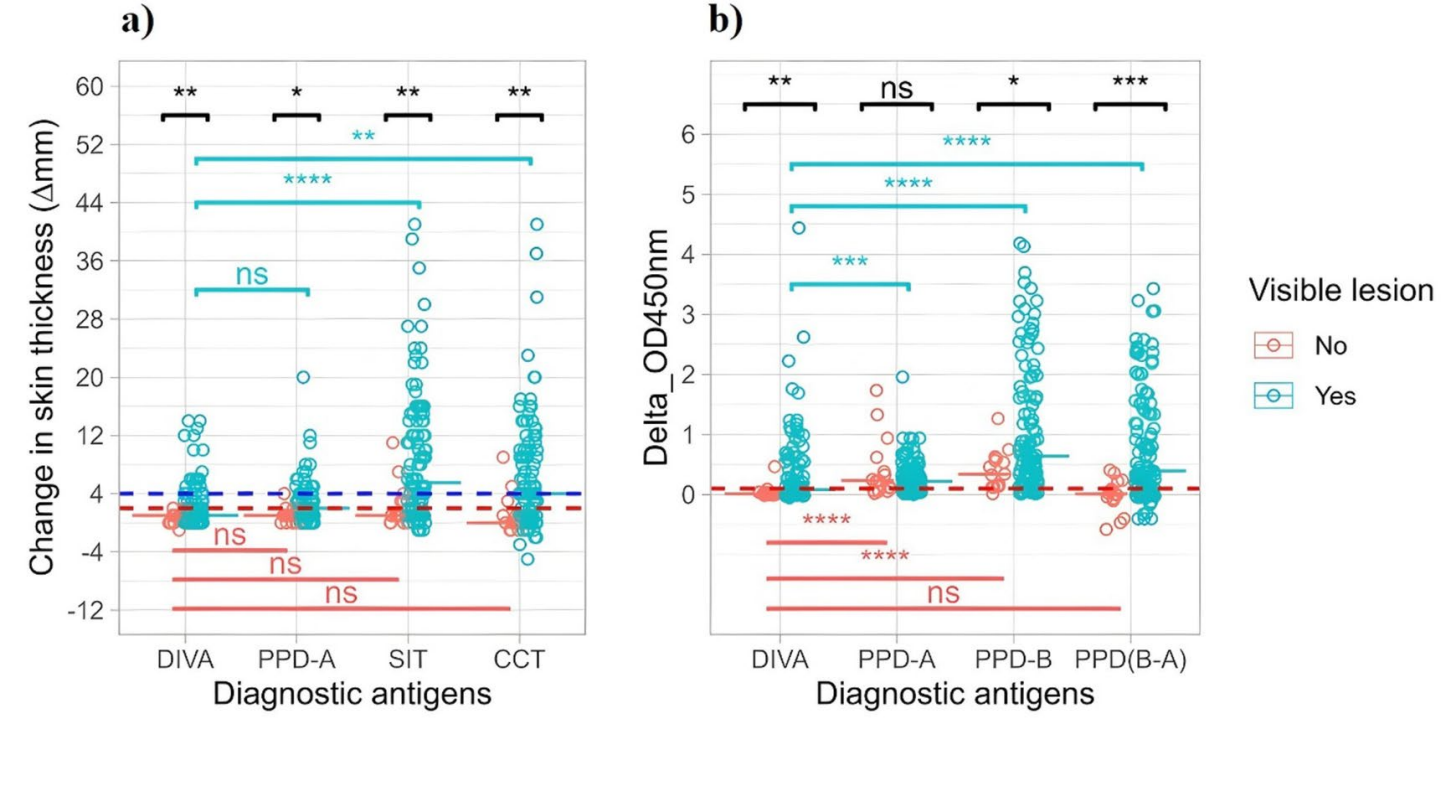
Twelve-month postexposure immune response to tuberculin and DIVA antigens. (**a**) Change in skin thickness and (**b**) IFN-γ response to DIVA and tuberculin in **65** unvaccinated control and **62** BCG-vaccinated calves exposed to natural infection for a year. Skin and IFN-γ test responses to DIVA and tuberculin were evaluated after 12 months of exposure to infected herds (14 months after BCG vaccination). The dotted blue horizontal lines at 4 mm and the dotted red horizontal lines at 2 mm are the cutoffs used for tuberculin and DIVA skin test antigens, respectively. The dotted red horizontal line at 0.1 is the cutoff used for all tuberculin and DIVA IFN-γ responses. The horizontal line within the plots of animals with and without visible lesions represents the median change in response to all the respective antigens. The statistical significance differences in skin reactions as well as IFN-γ responses between different antigens within the lesioned and non-lesioned groups were determined using Friedman repeated measures nonparametric analysis of variance with Bonferroni correction for multiple comparisons. The statistical significance of differences between the animals with and without visible lesions was determined using the Wilcoxon rank sum (Mann‒Whitney U) test (****P < 0.0001; ***P < 0.001; **P < 0.01; *P < 0.05; *ns* not significant (*P* > 0.05)).

The results of the Mann‒Whitney U test indicated that skin and IFN-γ responses to all antigens were significantly greater (*P* > 0.05) in animals with visible lesions, except for the IFN-γ response to avian tuberculin (*p* = 0.69) (Fig. 3a and b). The rank biserial correlation coefficient (r), which indicates the effect size, showed very large differences for all antigens. Tuberculin antigens elicited significantly greater skin and IGRA responses than DIVA antigens in animals with visible lesions (*p* < 0.0001) (Fig. 3a). Friedman repeated measures nonparametric ANOVA, with a Bonferroni correction for multiple comparisons, demonstrated a significantly greater skin test response in the SIT (*p* < 0.0001) and CCT (*p* < 0.01) tests than in the DIVA skin test while the skin test responses to avian tuberculin and DIVA were equivalent (*p* > 0.05). However, there were no significant differences in the skin test responses to any of the tuberculin or DIVA antigens (*p* = 0.07) among the animals without visible lesions. In contrast, both avian and bovine tuberculin antigens induced significantly greater IFN-γ responses than DIVA IGRA (*p* < 0.0001) in the animals without visible lesions, while the bovine minus avian tuberculin and DIVA IGRA test responses were comparable (*p* > 0.05) (Fig. 3b).

### Sensitivity of skin tests and IFN-γ assays compared against *M. bovis* culture PCR test

The performance measures of all skin and IGRA test antigens evaluated at the 12th month postexposure in the BCG-vaccinated and unvaccinated animals were presented in Table 3. The findings indicate that the CCT at the standard cutoff (> 4 mm), DIVA skin test at cutoff (≥ 2 mm), and DIVA IGRA tests show comparable levels of accuracy and performance, albeit with an overall relative sensitivity of less than 50%. At the end of the exposure period, out of 47 culture PCR-positive unvaccinated cattle, 25 and 23 had positive skin fold thickness responses to the DIVA and CCT skin tests, respectively, whereas out of 42 culture PCR-positive BCG-vaccinated group, 16 and 17 animals had positive skin reactions to the DIVA and CCT skin tests, respectively (Table 3). The results indicate that the DIVA and CCT have overall relative sensitivities to culture PCR status of 46% (95% CI 36, 56) and 45% (95% CI 35, 55), respectively. The DIVA IGRA test demonstrated an overall relative sensitivity of 47% (95% CI 37, 57). On the other hand, both the bovine and bovine minus avian tuberculin IGRA tests with the SIT skin test demonstrate comparable accuracy and performance, with significantly greater overall sensitivity of over 60% compared to CCT, DIVA skin, and DIVA IGRA tests regardless of the treatment group. Although the relative sensitivities of all tests were slightly greater in the unvaccinated control calves than in the BCG-vaccinated calves, these differences were not statistically significant. The sensitivity of all skin tests and IFN-γ assays was similar, with no statistically significant differences, when evaluated against the combined visible lesions or *M. bovis* PCR status reference standard (Supplementary Table S2) compared to the *M. bovis* culture PCR status reference (Table 3).

**Table 3:** Relative sensitivity of IGRA and skin tests with respect to *M. Bovis* culture PCR positivity in BCG vaccinated and unvaccinated control animals at the 12th month postexposure.

Treatment	Test	Antigen	Cutoff	Status	*M. bovis* Culture PCR		Sensitivity (95% CI)	LR+	LR−	Accuracy
					Positive	Negative				
BCG vaccinated	Skin	DIVA	≥ 2 mm	Positive	16	8	38 (25, 53)	0.95	1.03	0.45
				Negative	26	12				
		CCT	> 4 mm	Positive	17	6	40 (27, 56)	1.35	0.85	0.50
				Negative	25	14				
		SIT	≥ 4 mm	Positive	27	7	64 (49, 77)	1.84	0.55	0.65
				Negative	15	13				
	IGRA	DIVA	≥ 0.1	Positive	16	5	38 (25, 53)	1.52	0.83	0.50
				Negative	26	15				
		PPD (B-A)	≥ 0.1	Positive	29	11	69 (54, 81)	1.26	0.69	0.61
				Negative	13	9				
		PPD-B	≥ 0.1	Positive	38	17	90 (78, 96)	1.06	0.63	0.66
				Negative	4	3				
Unvaccinated controls	Skin	DIVA	≥ 2 mm	Positive	25	7	53 (39, 67)	1.37	0.77	0.55
				Negative	22	11				
		CCT	> 4 mm	Positive	23	5	49 (35, 63)	1.76	0.71	0.55
				Negative	24	13				
		SIT	≥ 4 mm	Positive	31	10	66 (52, 78)	1.19	0.77	0.60
				Negative	16	8				
	IGRA	DIVA	≥ 0.1	Positive	26	3	55 (41, 69)	3.32	0.54	0.63
				Negative	21	15				
		PPD (B-A)	≥ 0.1	Positive	39	9	83 (70, 91)	1.66	0.34	0.74
				Negative	8	9				
		PPD-B	≥ 0.1	Positive	42	17	89 (77, 95)	0.95	1.91	0.66
				Negative	5	1				

### Correlations between antemortem diagnostic tests of bTB

**Fig. 4:**
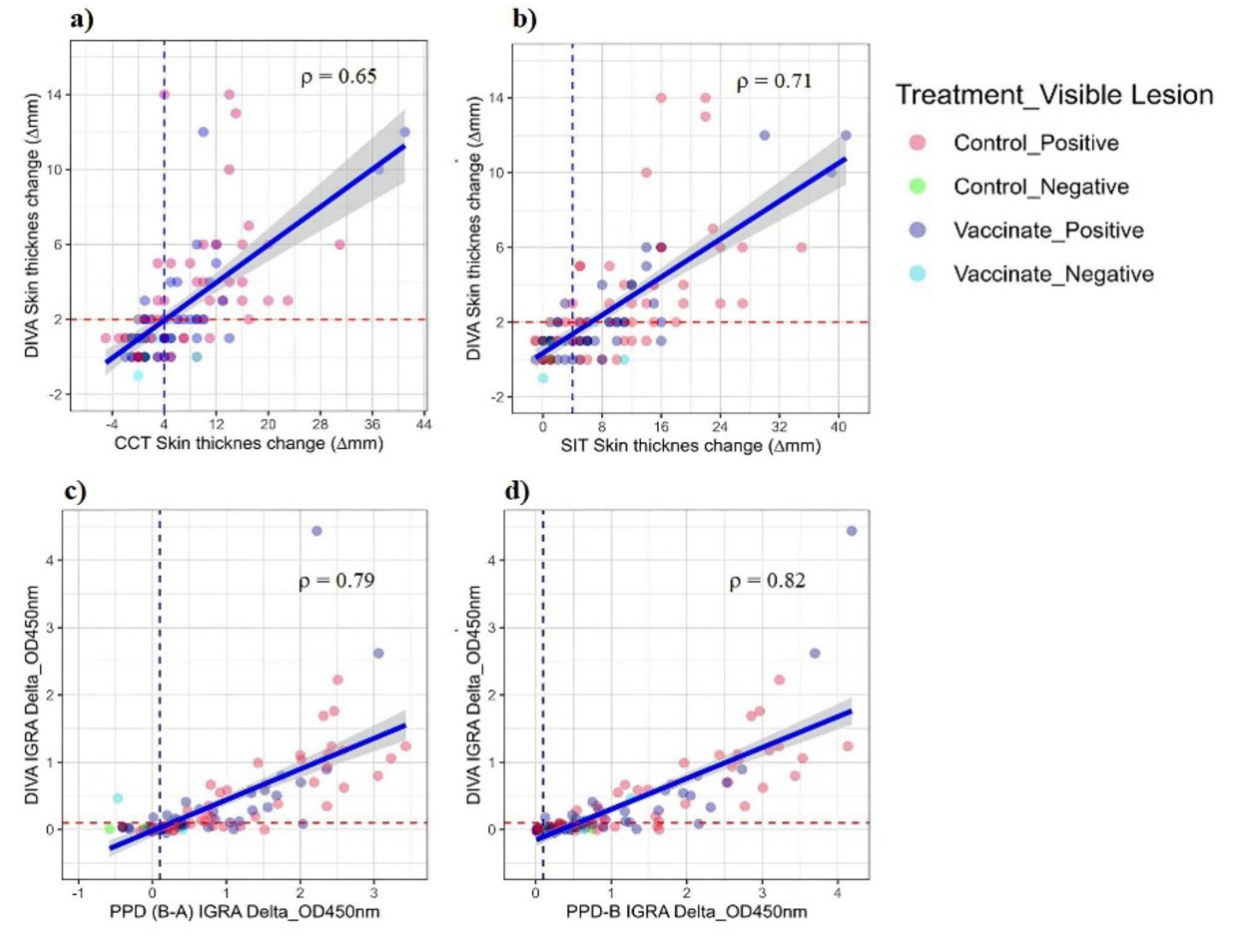
Correlation between OD450 nm levels of IFN-γ and skin test responses (**a**) Between the DIVA skin and CCT skin tests. (**b**) Between the DIVA skin and SIT skin tests. (**c**) Between the DIVA IGRA and bovine minus avian tuberculin IFN-γ tests. (**d**) Between the DIVA IGRA and bovine tuberculin IFN-γ tests. The solid-colored circles represent individual animals with treatment and visible lesions status combinations. The dashed vertical and horizontal lines at 0.1 are the cut-offs used for tuberculin and DIVA IGRA IFN-γ responses. The dashed vertical and horizontal lines at 4 and 2 are the cut-offs used for the skin thickness in the SIT/CCT and DIVA skin test responses, respectively.

The correlation between skin and IFN-γ responses to DIVA and tuberculin antigens were evaluated using the nonparametric Spearman’s rank correlation coefficient (ρ). The DIVA skin test response was strongly correlated with the CCT (Spearman’s ρ = 0.65, *p* < 0.0001) and SIT (Spearman’s ρ = 0.71, *p* < 0.0001) tuberculin skin tests responses. Similarly, the DIVA IFN-γ test responses were strongly positively correlated with both the bovine minus avian tuberculin (Spearman’s ρ = 0.79, *p* < 0.0001) and the bovine tuberculin IFN-γ test responses (Spearman’s ρ = 0.82, *p* < 0.0001). The correlation between DIVA skin and DIVA IFN-γ test responses was also strong and positive (Spearman’s ρ = 0.73; 95% CI 0.64–0.81, *p* < 0.0001), as was that between the CCT skin and bovine minus avian tuberculin IFN-γ responses (Spearman’s ρ = 0.79; 95% CI 0.71–0.85, *p* < 0.0001) (see Supplementary Fig. S2). All the above correlations were statistically significant and had large effect sizes, suggesting that both DIVA and tuberculin antigens used either in skin or IGRA induce similar immunological responses in bTB-infected animals (Fig. 4).

### Overlapping and distinct responses to multiple skin and IGRA test antigens

**Fig. 5:**
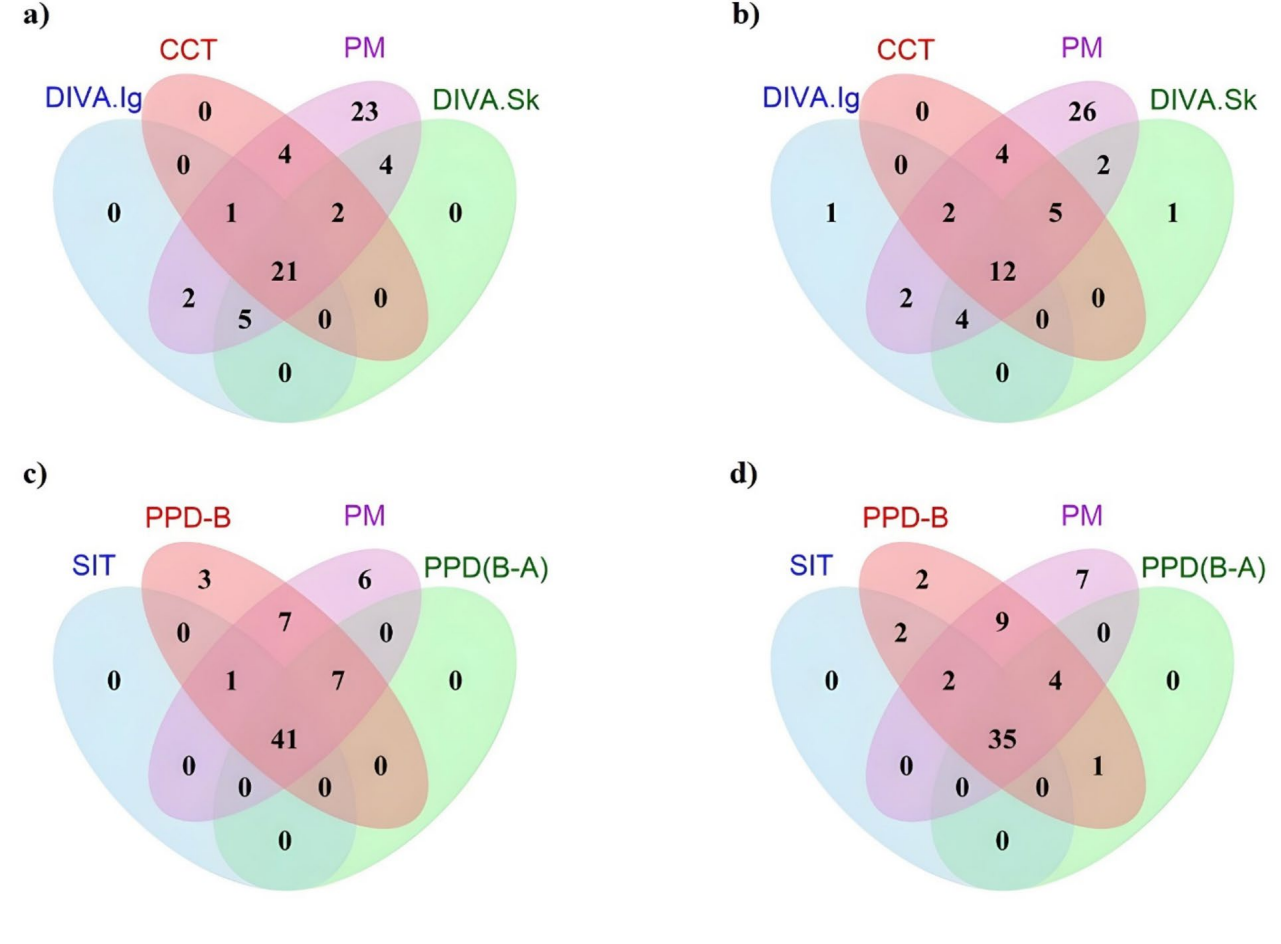
Venn diagram comparing the number of cattle positives for postmortem visible lesions, culture or *M. bovis* PCR (PM) with the number of (**a**) unvaccinated control and (**b**) vaccinated cattle tested positive on the DIVA IGRA (DIVA.Ig), CCT or DIVA skin test (DIVA.Sk) (**c**) unvaccinated and (**d**) vaccinated cattle tested positive on the SIT skin test, PPD-B or PPD (B-A) IGRA tests. All numbers within the purple circle represent animals that tested positive in the postmortem test (62 unvaccinated and 57 BCG vaccinated). All numbers outside the purple circle represent animals that tested negative in the postmortem test (3 unvaccinated controls and 5 BCG vaccinated).

All the three tests: DIVA IGRA, DIVA skin and CCT tests are concordant on 69% (88/127) of all tested animals at the end of the exposure period (Fig. 5 and Supplementary Table S3). The BCG-vaccinated animals had concordant outcomes for 74% (46/62) of animals using the DIVA IGRA and CCT skin tests. The CCT tuberculin skin and DIVA skin tests showed concordant test responses in 86% (56/65) of controls and 82% (51/62) vaccinated animals. Between the DIVA IGRA and DIVA skin tests, a similar concordance of 86% (56/65) was observed in unvaccinated controls while 79% (49/62) was seen in BCG vaccinated cattle (Fig. 5 and Supplementary Table S3). The bovine minus avian tuberculin IGRA and DIVA IGRA test results are concordant in 71% (46/65) of controls and in 63% (39/62) of vaccinated animals (Supplementary Fig. S3 and Table S4). Diagnostic status based on the DIVA IFN-γ and DIVA skin test formats showed substantial agreement, with observed agreement on 83% of the cases and a kappa value of 0.645 (95% CI 0.51, 0.78), suggesting a level of concordance beyond that expected by chance. The bovine tuberculin and bovine minus avian tuberculin IGRA tests and bovine tuberculin skin test (SIT) are concordant on 70% (89/127) of the animals. In the unvaccinated control group, animals that tested positive for all test antigens except for bovine tuberculin IGRA were positive in postmortem tests (lesions, culture, or PCR). However, the bovine tuberculin IGRA test showed positive results in all postmortem test-negative unvaccinated and BCG-vaccinated animals. In BCG-vaccinated cattle, the DIVA skin test, DIVA IGRA test, and bovine minus avian tuberculin IGRA each tested positive in one postmortem test negative animal. Additionally, the bovine tuberculin skin test was positive in two postmortem test negative cattle.

## Discussion

Incorporating BCG vaccination into control strategies has the potential to offer a cost-effective and sustainable method of controlling bTB in regions where the “test and cull” strategy might not be feasible or persistent wildlife reservoirs pose challenges. Studies show that BCG vaccination reduces bTB pathology and transmission in cattle^7,8,10^. BCG, the most widely used vaccine against human tuberculosis is approved for some wildlife species such as the Eurasian badger^1,38^. However, deployment of BCG vaccination to control bTB in cattle requires validation of a diagnostic assay with reliable sensitivity, specificity, and DIVA capability.

This study evaluated immune responses to tuberculin and DIVA peptide cocktails (ESAT-6, CFP10, and Rv3615c) in IFN-γ and skin tests in 67 BCG-vaccinated and 67 unvaccinated controls prior to exposure and in 65 unvaccinated and 62 BCG-vaccinated cattle after one year of exposure to natural infection. Eight weeks post BCG, all DIVA skin and IGRA tests as well as all tuberculin skin tests demonstrated 100% specificity in the unvaccinated control calves. In vaccinated calves, DIVA IGRA and skin tests had specificities of 98.5% and 94.0%, respectively. All DIVA skin and IGRA test positive calves at preexposure were BCG-vaccinated that showed responses close to the marginal cutoff values in their respective tests. However, upon retesting at the 4th and 2nd month post-exposure, respectively, they tested negative in these same tests except one. This suggests that the initial marginally positive test responses before exposure were likely false positives. This aligns with previously reported lower proportions of positive DIVA IFN-γ responses in BCG-vaccinated calves^24^ and positive DIVA skin test responses in Gudair-vaccinated calves^39^. However, it is difficult to explain why such erratic positive responses to DVIA antigens in both skin and IGRA tests occur in vaccinated calves.

In contrast to DIVA, tuberculin antigens elicited significantly higher skin and IFN-γ responses eight-weeks post-BCG in vaccinated cattle. This elevated pre-exposure response is as expected given that the DIVA antigens were selected specifically not to elicit an immune response in BCG vaccinates in contrast to tuberculin that contains a broader range of antigens that can cross-react with BCG. Several previous studies have also reported a higher skin thickness response to tuberculin than to DIVA antigens in infected cattle with lesions^12,13,17,39,40^ and greater IFN-γ response to tuberculin compared to ESAT-6 in *M. bovis* culture-positive cattle than in *M. bovis* culture-negative animals^39,41^. A positive bovine minus avian as well as bovine tuberculin IFN-γ response was also observed in unvaccinated control calves at eight-weeks post BCG and in vaccinated cattle without visible lesions at the end of exposure. Positive tuberculin IFN-γ response in unvaccinated control at preexposure may have been due to nonspecific immune response to environmental mycobacterial exposure. The IFN-γ responses biased towards avian tuberculin, suggest sensitization to environmental mycobacteria. This further suggests that the tuberculin antigens lack specificity in regions where animals are possibly exposed to environmental mycobacteria, leading to immune sensitization. The observed nonspecific positive IFN-γ test response to tuberculin in unvaccinated and noninfected control cattle in the current is consistent with previous reports^15,19^. Young cattle are reported to have more NK cells and exhibit nonspecific IFN-γ production in as much as one-third of the calves^42,43^. A strong avian tuberculin-biased response has also been reported in Gudair-vaccinated and noninfected control cattle^39^.

The DIVA skin and IFN-γ responses were significantly lower than those of tuberculin; however, DIVA tests performed comparably to the comparative cervical test (CCT). These three tests showed less than 50% sensitivity in cattle that were confirmed positive for *M. bovis* in culture PCR or through the presence of lesions indicative of possible infection. In contrast, the bovine and bovine minus avian tuberculin IGRA tests, as well as the SIT skin test, showed significantly higher sensitivities of about 60% and above in the same groups compared to the CCT, DIVA skin, and DIVA IGRA tests. However, these tests are less specific in unvaccinated controls exposed to environmental mycobacteria and BCG-vaccinated cattle. More than 20% of animals with a positive immune response to all skin and IGRA tests, indicating possible infection, were *M. bovis* culture PCR-negative. This suggests a lack of sensitivity in mycobacterial culture for detecting potentially infected cattle. Three controls and five vaccinates (100%) of claves that tested negative in all postmortem tests (bTB lesions, culture, or PCR) also tested positive in the bovine tuberculin minus nil IGRA test (Fig. 5). This demonstrates the lack of specificity of the bovine tuberculin in the IGRA test. This lack of specificity could be due to environmental mycobacterial exposure or the effect of BCG, which suggests that using the IGRA bovine tuberculin alone in areas with a high likelihood of exposure to environmental mycobacteria is inappropriate.

Previous research reported DIVA skin and IGRA test sensitivities higher than 63% in experimentally and naturally infected tuberculous cattle^19,39^. Several factors, including regional differences related to herd size, breed, age, stage of disease, repeated tuberculin skin testing, the prevalence of environmental mycobacteria, helminth infections, and pathogens other than mycobacteria causing granulomas, might have contributed to the variations and lower relative sensitivity observed in our study. Repeated tuberculin skin tests have been reported to cause skin desensitization in naturally infected cattle, leading to a lower overall sensitivity^44,45^. We also noticed a decrease in the amplitude of the skin test response in the terminal test conducted for the fourth repeated time (see Supplementary Fig. S2). Despite regular deworming, 22% of our study animals had an active liver fluke infection with a lower mean total lesion score compared to animals without active fasciolosis postmortem, suggesting a negative effect of active fasciolosis on lesion severity (*p* = 0.07). A reduced magnitude of skin response to tuberculin has been reported in cattle co-infected with tuberculosis and liver flukes^46^. In a study conducted in Ireland, animals with fewer than 3.6 lesions at slaughter exhibited no reaction to the tuberculin skin test^47^.

Our study found a strong significant correlation between the DIVA peptide cocktail and tuberculin antigen responses in skin fold thickness increase and ΔOD450 nm values of IFN-γ responses. This finding agrees with the previously reported high correlation between DIVA- and tuberculin-induced skinfold thickness and IFN-γ OD450 nm^41,48^. Although the correlation does not indicate accuracy, a significant correlation between the immune responses elicited by DIVA and tuberculin antigens in the skin and IGRA tests suggests that both may measure the same underlying condition. The DIVA skin or DIVA IFN-γ tests used in combination with CCT yielded concordant test outcomes in approximately three-quarters or more of the same individual controls and vaccinated animals. The similar responses to these three tests, regardless of vaccination status, further suggest that the tests are consistent across different treatment groups and have comparable performances in measuring similar underlying disease status.

The limitations of this study include the use of diagnostic data collected to evaluate the efficacy of BCG through a natural transmission experiment^10^, that was not specifically designed for diagnostic test evaluation. Although Bayesian Latent Class Analysis is recommended for evaluating measures of diagnostic performance when a gold standard is not available, we used culture PCR test status as a reference standard to calculate the relative sensitivity. However, convergence of the standard Walter-Hui model for latent class analysis of diagnostic tests depends on having a set of at least two diagnostic test results for individuals from two populations with distinct prevalence. This latter requirement is not met for our data, limiting the value and reliability of these methods in this context.

The sensitivities of all the tests are not significantly different when using *M. bovis* culture PCR test status as a reference standard compared to using combinations of visible lesions and *M. bovis* culture PCR status as a reference (Table 3, Supplementary Table S3, Table S5, Supplementary Fig. S4). This suggests that the performance of these tests in detecting positive cases remains consistent, regardless of whether the reference standard is culture PCR or a combination of visible lesions and culture PCR test results. However, both tests used as a reference standard are imperfect to be considered as a true gold standard test since lesions are nonspecific and culture is less sensitive. Test sensitivity can vary depending on different epidemiological contexts influenced by breed, immunological status, co-infections, and regional strain differences. Therefore, the relative sensitivity of the tests reported in this study may not accurately reflect the performance of the tests in the field under varying epidemiological conditions.

Overall, our results confirm that DIVA antigens can be used to detect tuberculosis infection in BCG-vaccinated cattle with high specificity and sensitivity comparable to that of CCT. Thus, the DIVA antigens used in both IGRA and skin tests hold a promise for practical implementation alongside BCG vaccination. This approach is particularly relevant in areas where the test-and-slaughter strategy is not economically feasible and there is no alternative implementable bTB control option in resource-poor countries such as Ethiopia.

In general, these findings underscore the low sensitivity of the current antemortem bTB diagnostic tests and the need for more sensitive diagnostic tests, which are crucial for effective bTB control. In a population with a high prevalence of bTB, a test and slaughter strategy using low sensitivity tests is not expected to be effective in controlling the disease. Both the mean, and median bTB prevalence exceed 30%, in large and medium-sized herds in Central Ethiopia^4^. When a high prevalence of bTB is observed within a herd in some high-income countries, all animals in the infected herd are culled. In countries with lower bTB prevalence and more financial resources, total culling may be feasible and effective in eradicating the disease. However, in Ethiopia, the combination of high bTB prevalence, economic limitations, and logistical constraints makes total culling impractical. Culling the entire herd presents several significant challenges including difficulties in obtaining replacement herds, farmers’ reluctance to cull high milk-producing cows, and financial constraints associated with compensating for the loss of numerous dairy cattle. Given these challenges, vaccination presents a more viable and economically feasible alternative to total culling.

Therefore, BCG vaccination of newborn calves and testing replacement livestock before purchasing should be considered important alternative measures to prevent the transmission and spread of the disease. Farmers are also more likely to accept and participate in vaccination programs than culling programs.

## Electronic supplementary material

Below is the link to the electronic supplementary material.

Supplementary Material 1

## Data Availability

All the data and the codes are available at: https://github.com/afromsa/bTBDiagnosticTestsPerformanceMeasures.
