# Supplementary material for: Comparative performance of tuberculin and defined-antigen cocktails for detecting bovine tuberculosis in BCG-vaccinated cattle in natural settings
Source: Sci Rep. 2025 Feb 7;15:4564. doi: 10.1038/s41598-025-85389-1 (PMC11802902; doi:10.1038/s41598-025-85389-1)
Supplement: Supplementary file 1 — Supplementary Material 1 [file 41598_2025_85389_MOESM1_ESM.pdf]

SUPPLEMENTARY TABLES AND FIGURES

Tables S1 and S2, S3 and S4

Fig S1, S2, S3 and S4

Comparative performance of tuberculin and defined-antigen cocktails for detecting bovine tuberculosis in BCG-vaccinated cattle in natural settings

Authors: Abebe Fromsa[1,2*], Andrew J.K. Conlan[3*], Sreenidhi Srinivasan[4,5,6], Balako Gumi[1], Wegene Bedada[7], Miserach Zeleke[1], Dawit Worku[1], Matios Lakew[7], Biniam Tadesse[7], Berecha Bayissa[1‡], Asegedech Sirak[7], Musse Girma Abdela[1], Getnet Abie Mekonnen[7], Tesfaye Chibssa[7], Maroudam Veerasami[8], Gareth J. Jones[9], H. Martin Vordermeier[10], Nick Juleff[112], James L.N. Wood[3], Gobena Ameni[1,11*], & Vivek Kapur[4,5*]

Affiliations:

[1] Aklilu Lemma Institutes of Pathobiology, Addis Ababa University; Addis Ababa, Ethiopia.

[2] College of Veterinary Medicine and Agriculture, Addis Ababa University, Bishoftu, Ethiopia.

[3] Disease Dynamics Unit, Department of Veterinary Medicine; University of Cambridge, United Kingdom.

[4] Huck Institutes of Life Sciences, The Pennsylvania State University, University Park, PA, USA

[5] Department of Animal Science, The Pennsylvania State University, University Park, PA, USA

[6] The Global Health Initiative, Henry Ford Health; Detroit, MI, USA.

[7] Animal Health Institute; Sebeta, Ethiopia.

[8] CisGen Biotech Discoveries Pvt Ltd, Chennai, India.

[9] Animal and Plant Health Agency, Weybridge, UK

[10] Technical Consultant and Independent Researcher, Woking, UK

[11] Department of Veterinary Medicine, College of Agriculture and Veterinary Medicine, United Arab Emirates University, United Arab Emirates

[12] The Bill & Melinda Gates Foundation; Seattle, WA, United States

*Corresponding authors. Email: abebe.fromsa@aau.edu.et, vxk1@psu.edu, ajkc2@cam.ac.uk, gobena.ameni@uaeu.ac.ae

‡Present address: National Veterinary Institute, Bishoftu, Ethiopia

Supplementary Table 1 (Table S1). 2 × 2 contingency table used to define measures of diagnostic test performance

Antemortem Test	Postmortem test		Row Totals
	Positive	Negative	
Positive	a (TP)	b (FP)	a + d
Negative	c (FN)	d (TN)	c + d
Column Totals	a + c	b + d	(a + b + c + d) = N

TP = True positive, FN = False negative, FP = False positive, TN = True negative

Supplementary Table 2 (Table S2). Relative sensitivity and specificity of IGRA and skin tests with respect to visible lesion (VL), culture or PCR positivity in BCG vaccinated and unvaccinated control animals at the 12th month postexposure

**Treatment**	**Test**	**Antigen**	**Cutoff**	**Status**	***M. bovis* Culture PCR or Visible Lesions**		**Sensitivity (95%CI)**	**LR+**	**LR-**	**Accuracy**
					**Positive**	**Negative**				
BCG vaccinated	Skin	DIVA	≥ 2 mm	Positive	24	1	42 (30, 55)	2.11	0.72	0.45
				Negative	33	4				
		CCT	> 4 mm	Positive	23	0	40 (29, 53)	-	0.60	0.45
				Negative	34	5				
		SIT	≥ 4 mm	Positive	37	2	65 (52, 76)	0.4	0.59	0.65
				Negative	20	3				
	IGRA	DIVA	≥ 0.1	Positive	21	1	37 (26, 50)	1.84	0.79	0.40
				Negative	36	4				
		PPD (B-A)	≥ 0.1	Positive	39	1	68 (56, 79)	3.42	0.40	0.69
				Negative	18	4				
		PPD-B	≥ 0.1	Positive	50	5	88 (77, 94)	0.88	-	0.81
				Negative	7	0				
Unvaccinated controls	Skin	DIVA	≥ 2 mm	Positive	32	0	52 (49, 64)	-	0.48	0.54
				Negative	30	3				
		CCT	> 4 mm	Positive	28	0	45 (33, 57)	-	0.55	0.48
				Negative	34	3				
		SIT	≥ 4 mm	Positive	42	0	68 (55, 78)	-	0.32	0.69
				Negative	20	3				
	IGRA	DIVA	≥ 0.1	Positive	29	0	47 (35, 59)	-	0.53	0.49
				Negative	33	3				
		PPD (B-A)	≥ 0.1	Positive	48	0	77 (66, 86)	-	0.23	0.78
				Negative	14	3				
		PPD-B	≥ 0.1	Positive	56	3	90 (80, 95)	0.90	-	0.86
				Negative	6	0				

Supplementary Table 3 (Table S3). Summary of concordant test outcomes between antemortem bTB diagnostic tests to supplement the data presented in the Venn diagram in Figure 5

**DST skin, DST IGRA and CCT Tests**						**Concordance**				
	**PM or PCR+**		**PM or PCR -**		**Total**	**All three tests**		**Paired tests**		
**Treatment**	**Test+**	**Test-**	**Test+**	**Test-**	**Tested**	**Sum**	**Percent**	**DST IGRA & DST skin**	**DST IGRA & CCT skin**	**CCT & DST skin**
Controls	21	23	0	3	65	47	47/65 (72%)	56/65 (86%)	52/65 (80%)	56/65 (86%)
Vaccinates	12	26	1 +/- 1	3	62	41	41/62 (66%)	49/62(79%)	46/62 (74%)	51/62 (82%)
Total	33	49	1 +/- 1	6	127	88	88/127 (69%)	105/127 (83%)	98/127 (77%)	110/127 (87%)
**SIT, IGRA PPD (B-A) or PPD-B Tests**						All three tests		Paired tests		
**Treatment**	Test+	Test-	Test+	Test-	Tested	Sum	Percent	SIT & IGRA PPD (B-A)	SIT & IGRA PPD-B	PPD (B-A) & PPD-B IGRAs
Controls	41	6	0 or 3	0	65	47	47/65 (72%)	57/65 (88%)	48/65 (74%)	54/65 (83%)
Vaccinates	35	7	1, 2 or 5	0	62	42	42/62 (68%)	53/62 (85%)	46/62 (74%)	47/62 (76%)
Total	76	13	-	0	127	89	89/127 (70%)	110/127 (87%)	94/127 (74%)	101/127 (80%)

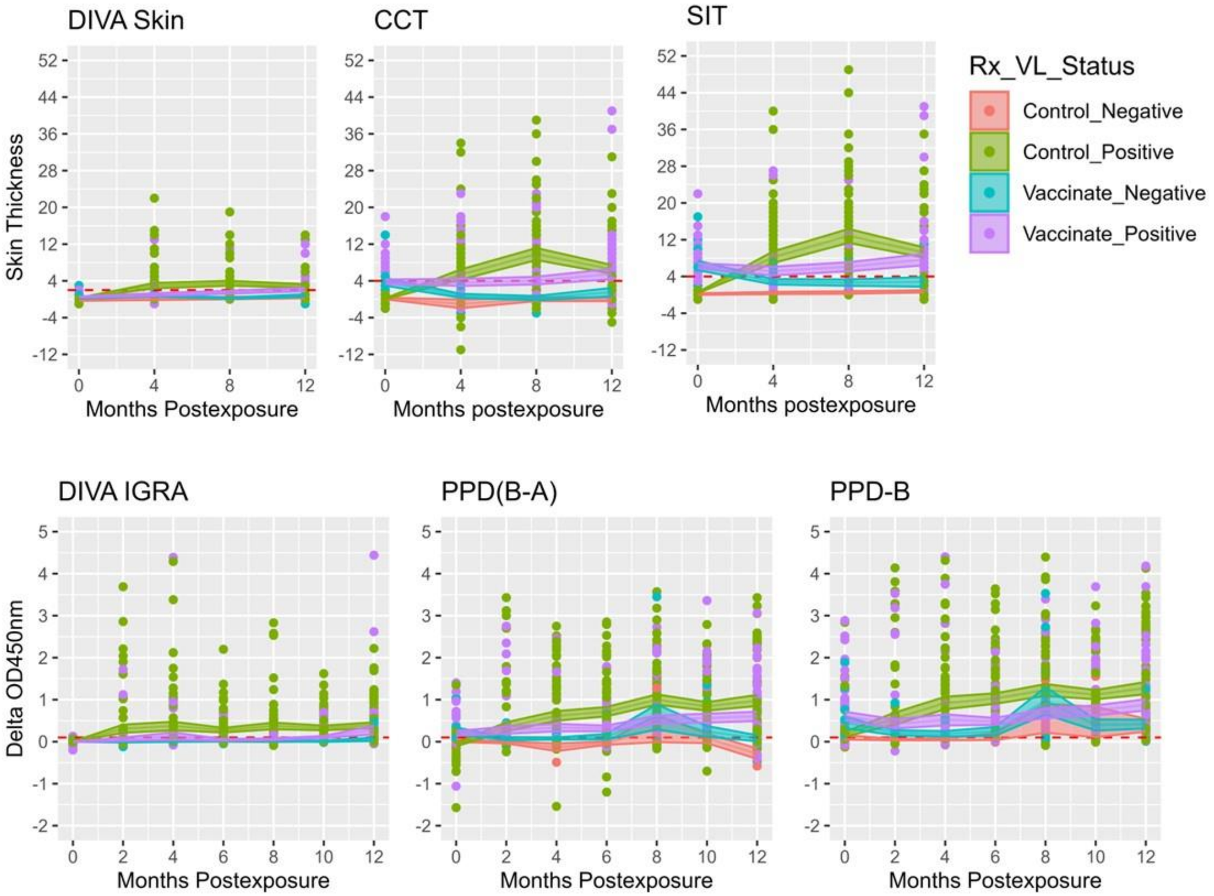

Supplementary Figure S1. Reduced skin test response at the fourth repeated test is suggestive of desensitization in the skin test (upper 3 panels) and not seen in the IGRA test results (lower 3 panels). The ribbon plots display the mean across all animals for each time point (line) and one standard error around the mean (shaded interval). Rx_VL_Status = Combination of treatment and visible lesion status of animals

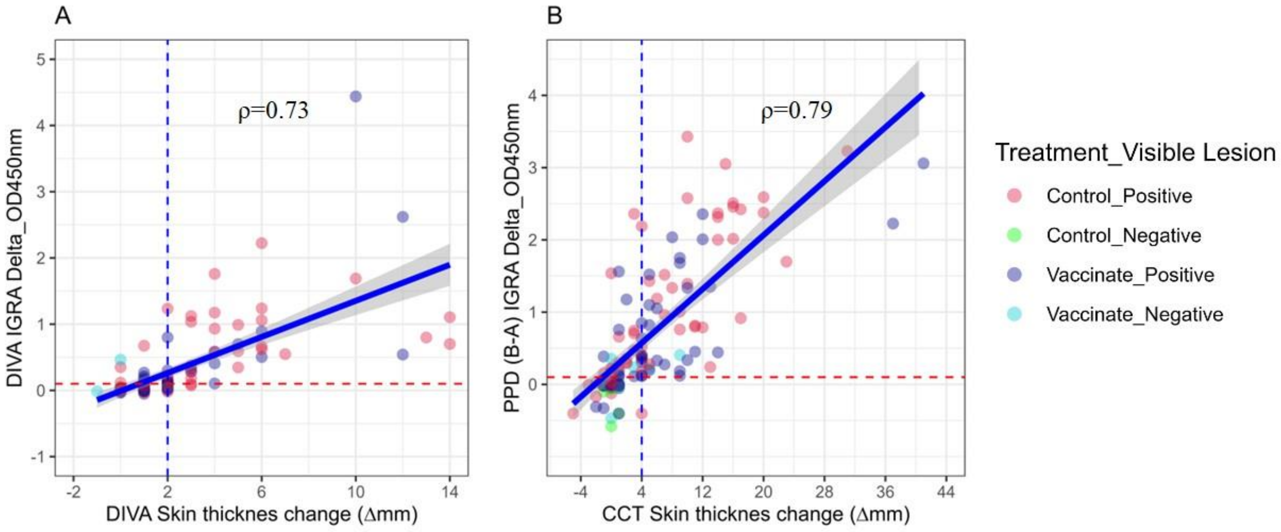

Supplementary Figure S2 (Figure S1). Correlation between OD450 nm levels of IFN-γ and skin test responses A) Between the DIVA skin and the DIVA IGRA tests. b) Between the CCT skin and bovine minus avian tuberculin IFN-γ tests. The solid-colored circles represent individual animals with treatment and visible lesions status combinations. The dashed horizontal red lines at 0.1 are the cut-offs used for tuberculin and DIVA IFN-γ responses. The dashed vertical blue lines at 2 and 4 are the cut-offs used for the DIVA and CCT skin thickness test responses, respectively.

Concordance of the skin and IGRA tests on visible lesions, culture or PCR test status

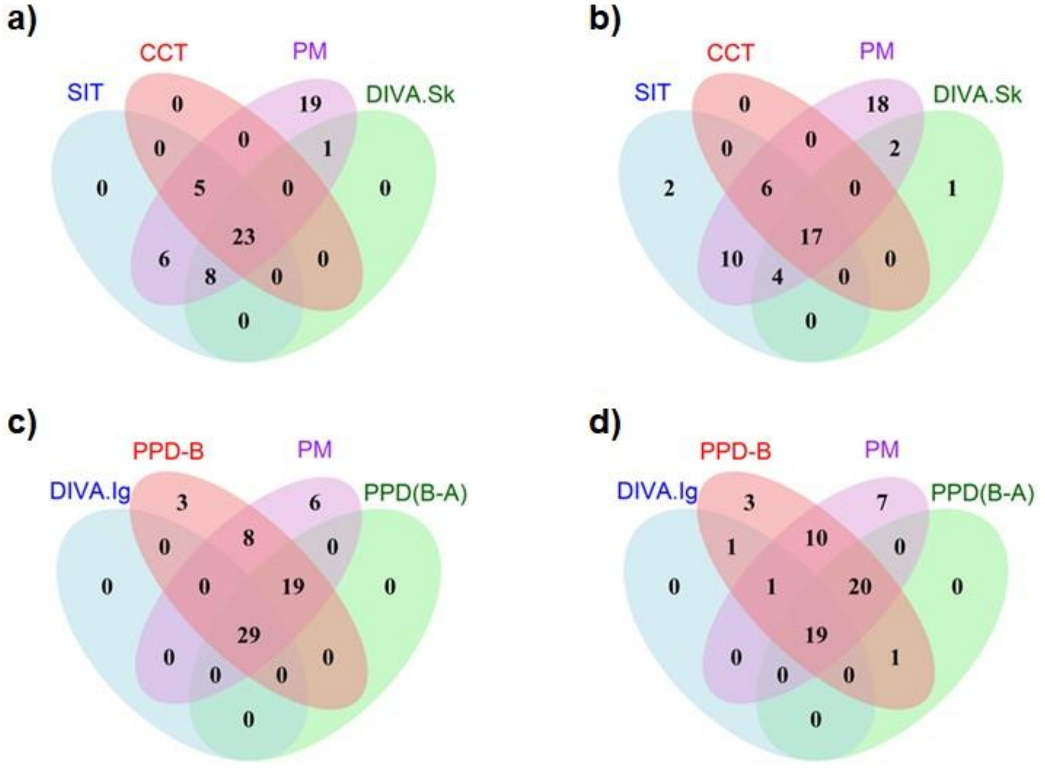

Supplementary Figure S3 (Figure S3). Venn diagram comparing the number of cattle positives for visible lesions, culture or *M. bovis* PCR (PM) with the number of (**a**) unvaccinated control and (**b**) vaccinated cattle tested positive on the skin test formats: DIVA skin (DIVA.Sk), CCT or SIT (**c**) unvaccinated and (**d**) vaccinated cattle tested positive on the IGRA test format: DIVA IGRA (DIVA.Ig), PPD (B-A) or PPD-B. All numbers within the purple circle represent animals that tested positive in the postmortem test (62 unvaccinated and 57 BCG vaccinated). All numbers outside the purple circle represent animals that tested negative in the postmortem test (3 unvaccinated controls and 5 BCG vaccinated).

Supplementary Table 4 (Table S4) to Supplementary Figure S3 (Figure S3).

	DIVA, CCT and SIT Skin Tests							Concordance		
	PM, culture or PCR+		PM, culture or PCR-		Total	All three skin tests		Paired tests		
**Treatment**	**Test+**	**Test-**	**Test+**	**Test-**	**Tested**	**Sum**	**Percent**	**DIVA & CCT**	**DIVA & SIT**	**CCT & SIT**
Controls	23	19	0	3	65	45	45/65 (69%)	56/65 (86%)	50/65 (77%)	48/65 (74%)
Vaccinates	17	18	3	2	62	37	37/62 (60%)	51/62 (82%)	39/62 (63%)	44/62 (71%)
**Total**	**40**	**37**	**3**	**5**	**127**	**82**	**82/127 (65%)**	**107/127 (84%)**	**89/127 (70%)**	**92/127 (72%)**
**DIVA, PPD (B-A) & PPD-B IGRA Tests**						**All three skin tests**		**Paired tests**		
**Treatment**	**Test+**	**Test-**	**Test+**	**Test-**	**Tested**	**Sum**	**Percent**	**DIVA & PPD (B-A)**	**DIVA & PPD-B**	**PPD (B-A) & PPD-B**
Controls	29	6	3	0	65	35	35/65 (54%)	46/65 (71%)	35/65 (54%)	54/65 (83%)
Vaccinates	19	7	4	1	62	27	27/62 (44%)	39/62 (63%)	28/62 (45%)	46/62 (74%)
**Total**	**48**	**13**	**7**	**1**	**127**	**62**	**62/127 (49%)**	**85/127 (67%)**	**63/127 (50%)**	**100/127 (79%)**

Concordance of the skin and IGRA tests on *M. bovis* PCR test status alone

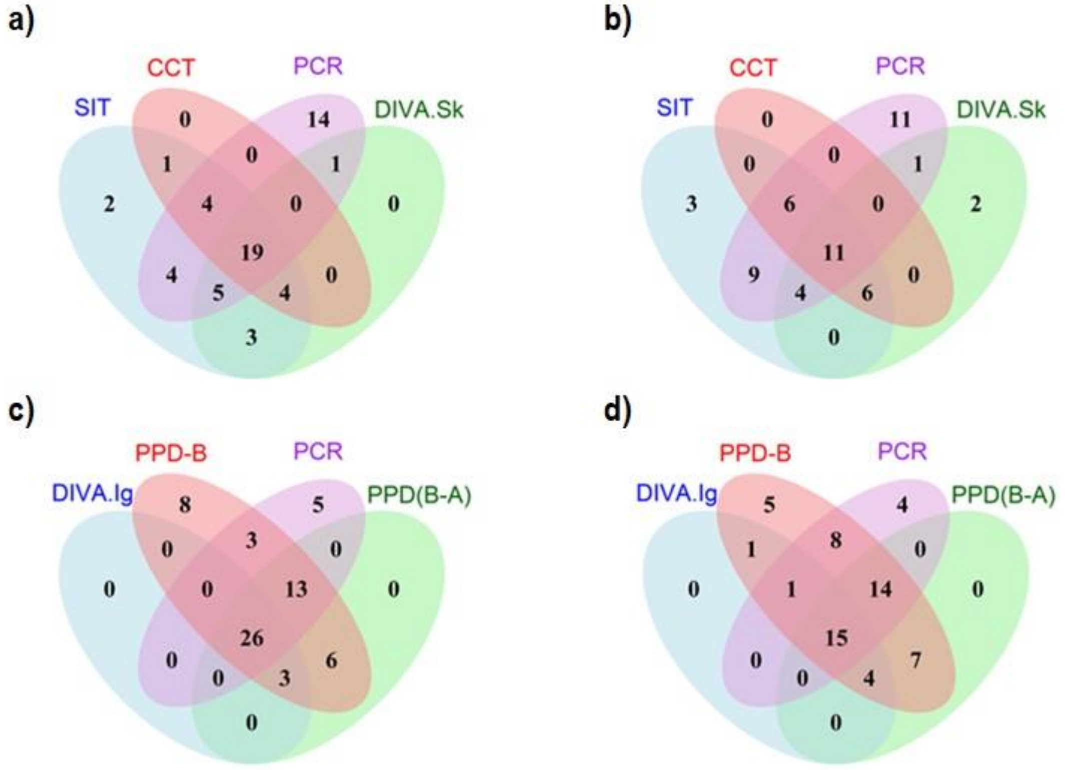

Supplementary Figure S4 (Figure S4). Venn diagram comparing the number of cattle positives for M. bovis culture PCR (PCR) with the number of (a) unvaccinated control and (b) vaccinated cattle tested positive on the skin test formats: DIVA skin (DIVA.Sk), CCT or SIT (c) unvaccinated and (d) vaccinated cattle tested positive on the IGRA test format: DIVA IGRA (DIVA.Ig), PPD (B-A) or PPD-B. All numbers within the purple circle represent animals that tested positive in M. bovis culture PCR (47 unvaccinated and 42 BCG vaccinated). All numbers outside the purple circle represent animals that were M. bovis culture PCR negative Note: The sum of M. bovis culture PCR negative animals outside the purple could be less than the maximum (18 unvaccinated controls and 20 BCG vaccinated) as they will be represented by 0 unless they are positive to the respective tests.

Supplementary Table 5 (Table S5) to Supplementary Figure S4 (Figure S4).

**DIVA, CCT and SIT Skin Tests**						**Concordance**				
	**PCR+**		**PCR-**		**Total**	**All three skin tests**		**Paired tests**		
**Treatment**	**Test+**	**Test-**	**Test+**	**Test-**	**Tested**	**Sum**	**Percent**	**DIVA &CCT**	**DIVA & SIT**	**CCT & SIT**
Controls	19	14	10	8	65	41	41/65 (63%)	40/65 (62%)	37/65 (57%)	43/65 (66%)
Vaccinates	11	11	11	9	62	31	31/62 (50%)	34/62 (55%)	28/62 (45%)	37/62 (60%)
**Total**	**30**	**25**	**21**	**17**	**127**	**72**	**72/127 (57%)**	**74/127 (58%)**	**65/127(51%)**	**80/127(63%)**
**DIVA, PPD (B-A) & PPD-B IGRA Tests**						**All three tests**		**Paired tests**		
**Treatment**	**Test+**	**Test-**	**Test+**	**Test-**	**Tested**	**Sum**	**Percent**	**DIVA & PPD (B-A)**	**DIVA & PPD-B**	**PPD (B-A) & PPD-B**
Controls	26	5	17	1	65	32	32/65 (49%)	45/65 (69%)	34/65 (52%)	44/65 (68%)
Vaccinates	15	4	17	3	62	22	22/62 (35%)	36/62 (58%)	26/62 (42%)	33/62 (53%)
**Total**	**41**	**9**	**34**	**4**	**127**	**54**	**54/127 (43%)**	**81/127 (64%)**	**60/127 (47%)**	**77/127(61%)**

Supplementary methods: Sample size determination

Our report represents a secondary analysis of data collected from a study where the primary objective was to estimate the efficacy of BCG vaccination to reduce transmission of bTB. For this primary objective the experimental reproduction ratio, defined as the expected number of new infections generated by a single infected animal over the course of the experiment, is the key design parameter. Given the chronic nature of bTB infection, R can be tuned by increasing (or decreasing) the duration of the experiment and thus the period of exposure between infectious and susceptible animals.

From previous studies we estimated that an in-contact period of 1 year would provide a long enough exposure to ensure an experimental R > 1 and produce sufficient infected seeder animals from Phase I to progress to Phase II (see https://doi.org/10.7554/eLife.27694 for full details). A conservative estimate of the sample size necessary to estimate a given total vaccine efficacy can be obtained using a final size method (https://doi.org/10.1017/S095026880600673X) for the total number of transmission events observed in the vaccinated and control groups. Considering each pair of susceptible and infected animals independently the probability of infection will approximately be:

$p_1 = \left(\frac{R_1}{R_1+2}\right)$ and $p_2 = \left(\frac{R_2}{R_2+2}\right)$

in the control and vaccinated groups respectively. R_1 and $R_2$are the experimental reproduction numbers in the control and vaccinated groups and $R_2 = \varepsilon_T R_1$ where the total vaccine efficacy to reduce transmission is $1 - \varepsilon_T$. The sample size can then be calculated using Fisher's exact test. When n is large (np > 0.5; n (1-p) > 5) the binomial distributions can be approximated by normal distributions leading to the following expression for n:

$$n = \big(p1(1-p) + p2\ (1-p2)\big)\left(\frac{Z_\propto + Z_\beta}{p_1 - p_2}\right)^2$$

Since we have two biologically replicated groups thus:

$$n = 2\big(p1(1-p) + p2\ (1-p2)\big)\left(\frac{Z_\propto + Z_\beta}{p_1 - p_2}\right)^2$$

Where:

- ✓ $Z_\propto$ *and* Z_β are the critical values of the standard Normal distribution for the two types of error, e.g. $Z_\propto$ = 1.96 for a 95% confidence level and Z_β = 0.842 (power 80%)

This method estimates that a group size of 52 animals (104 sentinel animals in total split between vaccinated and control groups) will provide 80% power to estimate an effect size (total vaccine efficacy) of 75% at the 95% significance level.

We aimed for a higher number (72 per group) than the calculated sample size required (72 per group) to account for potential losses and ensure robustness of the results. But when COVID-19 shutdown was declared, we were forced to start the experiment with the number of animals we already recruited that met the inclusion criteria, i.e. the 67 calves
